# Large epigenome-wide association study of childhood ADHD identifies peripheral DNA methylation associated with disease and polygenic risk burden

**DOI:** 10.1038/s41398-020-0710-4

**Published:** 2020-01-21

**Authors:** Michael A. Mooney, Peter Ryabinin, Beth Wilmot, Priya Bhatt, Jonathan Mill, Joel T. Nigg

**Affiliations:** 1 Division of Bioinformatics & Computational Biology, Department of Medical Informatics & Clinical Epidemiology, Oregon Health & Science University, Portland, OR USA; 2 OHSU Knight Cancer Institute, Portland, OR USA; 3 Oregon Clinical and Translational Research Institute, Portland, OR USA; 4 Division of Psychology, Department of Psychiatry, Oregon Health & Science University, Portland, OR USA; 5 University of Exeter Medical School, Exeter University, Exeter, UK; 6 Department of Behavioral Neuroscience, Oregon Health & Science University, Portland, OR USA

**Keywords:** Genomics, ADHD

## Abstract

Epigenetic variation in peripheral tissues is being widely studied as a molecular biomarker of complex disease and disease-related exposures. To date, few studies have examined differences in DNA methylation associated with attention-deficit hyperactivity disorder (ADHD). In this study, we profiled genetic and methylomic variation across the genome in saliva samples from children (age 7–12 years) with clinically established ADHD (*N* = 391) and nonpsychiatric controls (*N* = 213). We tested for differentially methylated positions (DMPs) associated with both ADHD diagnosis and ADHD polygenic risk score, by using linear regression models including smoking, medication effects, and other potential confounders in our statistical models. Our results support previously reported associations between ADHD and DNA methylation levels at sites annotated to *VIPR2*, and identify several novel disease-associated DMPs (*p* < 1e–5), although none of them were genome-wide significant. The two top-ranked, ADHD-associated DMPs (cg17478313 annotated to *SLC7A8* and cg21609804 annotated to *MARK2*) are also significantly associated with nearby SNPs (*p* = 1.2e–46 and *p* = 2.07e–59), providing evidence that disease-associated DMPs are under genetic control. We also report a genome-wide significant association between ADHD polygenic risk and variable DNA methylation at a site annotated to the promoter of *GART* and *SON* (*p* = 6.71E–8). Finally, we show that ADHD-associated SNPs colocalize with SNPs associated with methylation levels in saliva. This is the first large-scale study of DNA methylation in children with ADHD. Our results represent novel epigenetic biomarkers for ADHD that may be useful for patient stratification, reinforce the importance of genetic effects on DNA methylation, and provide plausible molecular mechanisms for ADHD risk variants.

## Introduction

Attention-deficit hyperactivity disorder (ADHD) has a substantial heritable component, with genetic factors interacting with early-life environmental exposures to mediate risk^1,2^. A recent large genome-wide association study (GWAS) meta-analysis of ADHD identified the first genome-wide significant association with common DNA variants^3^. These data allow for a more precise estimate of the burden of ADHD genetic risk, which is associated with ADHD diagnosis and some of its mechanisms, such as poor executive function^4^.

The specific mechanisms by which genetic risk factors influence ADHD are not known, although recent evidence supports a role for non-sequence-based (i.e., regulatory) genomic variation in neuropsychiatric phenotypes^5–8^. Epigenetic processes, which act to dynamically control gene expression and are known to regulate key neurobiological and cognitive processes in the brain, represent both a potential clue to mechanisms and a possible source of novel biomarker discovery^9,10^. Epigenetic studies of mental disorders have focused primarily on DNA methylation, the best-characterized and most stable epigenetic modification. It acts to influence gene expression via physical disruption of transcription factor binding and the attraction of methyl-binding proteins that initiate chromatin compaction and gene silencing^11^. Epigenetic variation can be influenced by environmental exposures. In fact, many of the environmental risk factors associated with ADHD have been associated with changes in DNA methylation in peripheral tissues^12–16^. Epigenetic changes may also be induced by exposures related to the disease (e.g., medication) or the pathological process itself. Although patterns of DNA methylation are cell-type specific, and generalization from peripheral tissue to brain mechanisms is not appropriate, disease-associated changes in peripheral DNA methylation might serve as useful biomarkers. For instance, DNA methylation changes in peripheral tissues may be indicative of early exposures (caused by, rather than causal of, disease process) or clinical subtypes, or may be predictors of outcome or treatment response^17^. Importantly, both genetic and environmental etiologies can operate through epigenetic effects, and there is evidence that genetic effects on DNA methylation are relatively conserved across tissues^18,19^. Thus, an integrated genetic/epigenetic approach is strongly recommended for “second generation” studies of methylation biomarkers^18,20^. Combining genetic and epigenetic data might also nominate novel mechanistic pathways associated with disease as hypotheses for future study^21^.

DNA methylation studies relevant to ADHD or ADHD symptoms to date are limited. They include five candidate gene studies on relatively small samples^22–26^, and four studies targeting specific environmental exposures, such as malnutrition, in relation to ADHD symptoms, by using candidate and epigenome-wide association study (EWAS) approaches^27–30^. Three prior EWAS studies are relevant. The first and only study to examine ADHD cases directly, was a small study by our group^31^. That study of 105 children was able to replicate suggestive findings in *MYT1L* and *VIPR2*, also a top hit in an EWAS of environmental exposure^28^ and a recent twin study^32^. An EWAS of ADHD symptoms in adult population cohorts reported additional novel candidate sites for exploration, as well as noting that most top-ranked loci were driven by DNA methylation quantitative trait loci (mQTL)^33^. A third population-based EWAS study^7^ identified prenatal DNA methylation sites related to later ADHD symptom trajectories, though they did not replicate at the age of 7.

We report here the first large-scale EWAS of children with ADHD and extend our previous study to incorporate analyses of genetic effects in the context of variable DNA methylation, exploring both mQTL and polygenic risk burden derived from GWAS. Given our focus on identifying biomarkers relevant to ADHD, we use DNA derived from saliva in an effort to minimize potential selection bias resulting from clinical differences in rates of child refusal of blood draws.

## Methods

### Participants and case identification

In a case-finding procedure, families were recruited by soliciting community volunteers with public advertisements and mass mailings. The local Institutional Review Board approved the studies. Parents provided written informed consent; children provided written informed assent. All families completed a multi-informant, multi-method screening process to establish eligibility and diagnostic group assignment for ADHD, non-ADHD, as well as comorbid disorders (Supplementary Materials).

### Medication

Current and lifetime prescription of any psychoactive medication, including any stimulant or non-stimulant preparation, was recorded and statistically controlled. A full frequency list of medications in the sample is given in Table S1.

### DNA methylation profiling

An overview of our data quality control (QC) and analysis workflow is provided in Fig. S1. Genomic DNA was isolated from saliva, bisulfite converted, and assessed for DNA methylation on the MethylationEPIC BeadChip (Illumina, Inc.) using a standard protocol. Raw data were imported into Genome Studio v2011.1 (Illumina, Inc.) to investigate sample hybridization quality and to extract signal intensities for each probe (Fig. S2). Data QC measures included manual inspection of beta distributions, curation of control probes using the Illumina BeadArray Controls Reporter, manual inspection of total CpG intensity distributions, sex prediction, outlier sample detection, and comparison of SNP probes on the MethylationEPIC with genotypes, using the *lumi*^34^ and *minfi*^35^ packages. Data were normalized with *lumi*^34^ using smooth quantile normalization. Cell-type profiles and proportions were calculated using reference-free cell-type prediction with the *RefFreeEWAS*^36^ package, and beta values were adjusted to account for cell-type proportions in each sample. Methylation values were set as missing if they deviated >4 times the interquartile range from the mean of each probe. Participant samples were further curated to restrict to unrelated children who met full criteria for non-ADHD or ADHD. We excluded a small number of children who only met criteria for the ADHD hyperactive profile, those with subthreshold ADHD (e.g., five symptoms), and those missing information about medication usage, resulting in a final sample of 604 unrelated children. For analyses examining the effects of the ADHD polygenic risk score, only children with European ancestry^4^ were included (*N* = 472). We removed probes for the following reasons (Table S2): detection call rate *p*-value is <0.01 in at least one sample, mapping to multiple genomic locations^37^, missing from the MethylationEPIC manifest, SNPs underlying the probe^37^, or non-autosomal probes. Our final dataset included 568,281 probes for analysis.

### Genotyping and polygenic score

Salivary DNA samples were genotyped, and the ADHD polygenic risk score (PRS) for each individual calculated as described previously^4^. Briefly, DNA was hybridized to the PsychCHIP_v1-1 (*N* = 603,132 SNPs), developed by Illumina, Inc. in collaboration with the Psychiatric Genetics Consortium (PGC). Genotypes were used to determine relatedness^38^ among samples and population stratification as previously described^4^. The PRS was constructed using the PGC + iPSYCH meta-analysis^3^ as the discovery dataset (details in Supplementary Materials).

### Analytic models and covariates

Differential global methylation (average methylation across all probes), as well as differentially methylated positions (DMPs) were evaluated using custom R scripts (R 3.5.0, https://github.com/pryabinin/ohsu_adhd_ewas). Briefly, linear models (Eqs. (1)–(5), below) were used to examine associations between DNA methylation and both ADHD status and ADHD PRS. All models used cell-type adjusted beta values^36^ as the outcome variable, and included covariates for sex, age (in years), the first three genomic principal components (PCs), medication usage (binary variable signifying the history of psychoactive or stimulant medication), and a maternal smoking score^12^, which are referred to below as the “standard covariates”. The first three genomic PCs were selected based on their association with self-reported ethnicity in our sample. The relationship between ethnicity and genomic PCs in our sample was verified using HapMap samples as a reference. In addition, for the PRS analysis, a covariate was included to account for the number of missing SNPs in the PRS calculation for each patient. Finally, due to sex differences in ADHD prevalence, salient clinical features^39^, and familial burden not explained by autosomal differences^40^, a sex interaction term was included for both ADHD status and ADHD PRS (Eqs. (4) and (5) to investigate sex-specific effects:

1 $${\mathrm{Mean}}\left( {\rm Beta}\,{\rm values}\left( {\rm adj} \right) \right)\sim 1 + {\mathrm{ADHD}}\,{\mathrm{status}} + {\mathrm{Standard}}\,{\mathrm{Covariates}}$$

2 $${\mathrm{Beta}\,\mathrm{value}}\left( {{\mathrm{adj}}} \right)\sim 1 + {\mathrm{ADHD}}\,{\mathrm{status}} + {\mathrm{Standard}}\,{\mathrm{Covariates}}$$

3 $${\mathrm{Beta}\,\mathrm{value}}\left( {{\mathrm{adj}}} \right)\sim 1 + {\mathrm{PRS}} + {\mathrm{Standard}}\,{\mathrm{Covariates}} + {\mathrm{Missing}}\,{\mathrm{PRS}\,\mathrm{SNPs}}$$

4 $${\mathrm{Beta}\,\mathrm{value}}\left( {{\mathrm{adj}}} \right)\sim 1 + {\mathrm{sex}} \ast {\mathrm{ADHD}}\,{\mathrm{status}} + {\mathrm{ADHD}}\,{\mathrm{status}} + {\mathrm{Standard}}\,{\mathrm{Covariate}}$$

5 $${\mathrm{Beta}\,\mathrm{value}}\left( {{\mathrm{adj}}} \right)\sim 1 + {\mathrm{sex}} \ast {\mathrm{PRS}} + {\mathrm{PRS}} + {\mathrm{Standard}}\,{\mathrm{Covariates}} + {\mathrm{Missing}}\,{\mathrm{PRS}\,\mathrm{SNPs}}$$

### Differentially methylated region analysis

Testing for differentially methylated regions (DMRs) was performed using the Comb-p software tool^41^, using the results of each regression model separately. The following Comb-p parameters were used: seed *p*-value = 0.001, maximum distance between probes = 500 bp, and a minimum of three probes allowed in a DMR. This set of parameters is consistent with previous work^42^ in the field and recommendations from simulation experiments^43^.

### DNA methylation quantitative trait loci analysis

DNA methylation quantitative trait loci (mQTLs) were calculated using a linear, additive model implemented in the GEM package in R^44^, where the methylation level at each DMP was regressed on the minor allele count of each SNP. Covariates for sex, age, and the first three genomic PCs were included in the model. For all DMPs with *p* < 1e–5 (34 total), all 6,374,797 autosomal SNPs were assessed (i.e., both *cis*- and *trans*-QTLs were tested; 216,743,098 tests).

### Colocalization analysis of mQTLs and ADHD-associated variants

DNA methylation QTLs discovered in our cohort were examined for evidence of colocalization with ADHD-associated variants. All pairs of methylation probes and SNPs within each of 12 ADHD-associated regions (Table S3), determined from the results of the recent PGC + iPSYCH ADHD GWAS meta-analysis^3^, were evaluated. Statistical analyses were performed using summary data-based Mendelian randomization^45^ (SMR), as well as the Bayesian colocalization method in the *coloc* package^46^ in R.

### Functional interpretation and gene set enrichment analyses

Downstream interpretation of the associated DMPs was performed using *Genetica* (Table S9), a custom R script developed in our lab (https://github.com/bhattp09/genetica) that collates information from publicly available databases (Supplementary Materials) and through the Weizmann Institute of Science (http://www.genecards.org/). Investigations into miRNA profiles were performed using the miRWalk tools^47^, and seed regions overlapping the hybridized probe sequence was confirmed using the UCSC Genome Browser^48^.

The *methylGSA* package^49^ was used to investigate whether DMPs were enriched among genes involved in a particular biological function or process. *methylGSA* implements an unbiased test of enrichment, accounting for the number of methylation probes within each gene. The default significance threshold suggested by *methylGSA* (*p* ≤ 0.001) was used to select DMPs for the enrichment analyses. We first tested for enrichment among brain-expressed genes, using two gene sets defined by the Human Protein Atlas^50^ (https://www.proteinatlas.org/humanproteome/tissue/brain): genes with brain-enriched expression (408 genes; five-fold higher expression in the brain compared with all other tissues), and genes with brain-elevated expression (1379 genes; five-fold higher expression in the brain compared with the average expression in all other tissues). We next tested for enrichment among all Gene Ontology (GO) categories. GO categories enriched with a false discovery rate-adjusted *p* ≤ 0.05 were considered significant.

### Multiple testing, significance thresholds, and statistical power

Within each EWAS analysis, DNA methylation sites were considered significantly differentially methylated if they met a Bonferroni-corrected *p*-value threshold of 8.8e–8, which is consistent with a recent recommendation for the genome-wide significance threshold for analyses of EPIC array data^51^. Following other studies, a p-value threshold of 1e–5 was used to identify suggestive DMPs for preliminary interpretation to guide future hypotheses^52^. A more inclusive *p*-value threshold of 0.001 was used to select DMPs for gene set enrichment analyses as suggested by the MethylGSA R package^49^. In the DMR analysis, a Šidák-corrected *p*-value < 0.05 was used to determine significance. Finally, a Bonferroni-corrected *p*-value threshold of 1.8e–10 was used to identify significant mQTLs.

The statistical power to detect an effect size of 1% difference in DNA methylation between cases and controls at a *p*-value < 8.8e–8 was calculated for all probes included in our analysis, following a previously described method^51^. Power calculations were done using each probe’s variance across all subjects, and the number for cases and controls in the experiment. The proportion of probes with a particular power threshold (ranging between 0 and 1) is shown in Fig. S3. Our study was well-powered (80%) to detect a 1% difference at ~68% of all sites interrogated.

## Results

### Sample overview and genome-wide DNA methylation variation

Table 1 provides a demographic and clinical description of the cohort. As intended, the ADHD group had significantly higher clinical symptoms, and as expected, had higher rates of psychiatric comorbidity, and more lifetime exposure to psychiatric medications. In the full sample, the ADHD group had slightly lower family income than controls (*p* = 0.03), but the difference was not significant in the European-ancestry subsample used for the PRS analyses. As is common in studies of ADHD, the ADHD group had a slightly lower IQ (WISC-IV FSIQ mean difference = 7.1; *p* = 5.6e–10), but both groups had mean IQ in the average range. The groups were of similar age, although age was included as a covariate as a precaution. Boys were overrepresented in the ADHD group and we adjusted all results for sex.

**Table 1:** Clinical and demographic description of sample.

	ADHD vs. control		ADHD polygenic risk score (European-ancestry only)	
	ADHD	non-ADHD	ADHD	non-ADHD
*N*	391	213	302	170
Age (years)	9.8 (1.4)	9.8 (1.4)	9.9 (1.4)	9.8 (1.4)
% male	71.6%*	51.60%	72.5%*	54.10%
% European ancestry	77.20%	79.80%	100.00%	100.00%
Family income ($K)	76.2 (40.7)*	84.1 (39.0)	80.2 (40.2)	85.7 (39.4)
Estimated full scale IQ	108.1 (13.7)*	115.2 (12.5)	109.2 (13.2)*	115.3 (12.7)
Inattention(T)	72.7 (12.1)*	44.5 (7.0)	73.0 (12.2)*	44.3 (7.0)
Hyperactivity-Imp(T)	67.8 (14.6)*	45.4 (7.5)	68.1 (14.7)*	45.0 (7.5)
Lifetime mood disorder	7.2%*	2.8%	7.6%*	3.0%
Lifetime anxiety disorder	19.9%*	8.5%	19.9%*	8.3%
Lifetime conduct disorder	1.3%	0.0%	1.3%	0.0%
Lifetime ODD	17.6%*	0.5%	19.5%*	0.0%
Ever psychiatric med	45.0%*	0.9%	48.3%*	0.0%

Medication history was defined as any lifetime use of psychiatric medication

Inattention is the inattention T score by parent-rated ADHD Rating Scale

Differences between ADHD/control groups were determined by Mann–Whitney U tests for continuous measures and Fisher’s exact tests for categorical measures

*ODD* oppositional defiance disorder, *Hyperactivity-Imp* Hyperactivity-impulsivity T score

Significant differences (*p* < 0.05) are indicated with an *

We calculated a predicted age for each child, based on age-associated DNA methylation sites^10^. As expected, reported ages and predicted ages were highly correlated (*r* = 0.58, *p* = 1.3e–54), providing reassurance as to the validity of the DNA methylation data obtained from saliva. There was no evidence for differential epigenetic age acceleration in the ADHD group (*p* = 0.98) (Fig. S4). We also tested for a “global” difference in DNA methylation levels between ADHD cases and controls, determined by averaging cell-type-corrected beta values across all 568,281 probes (see the “Methods” section, Eq. (1)), and found no significant difference (*p* = 0.74). This result indicates that, as expected, ADHD is not associated with any systemic methylomic differences across probes included on the Illumina EPIC array.

### DNA methylation associated with ADHD

Differential DNA methylation between ADHD cases and controls at individual sites was investigated by regressing methylation levels at each probe on ADHD status (Eq. (2)) with our standard covariates. EWAS results were well-controlled with an inflation factor *λ* = 1.02 (calculated as the ratio of the median observed log_10_(*p*-value) to the median expected log_10_(*p*-value))^53^. A Q–Q plot and Manhattan plot are presented in Fig. S5.

**Fig. 1:**
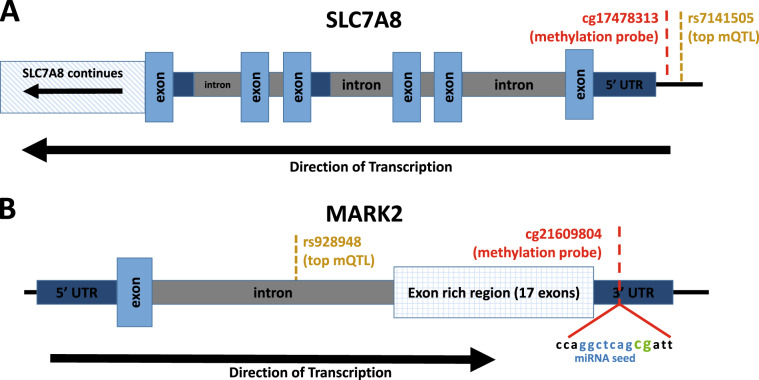
ADHD-associated DMPs in SLC7A8 and MARK2. Genomic diagrams of **a** SLC7A8 and **b** MARK2, showing locations of top DM probes and the corresponding mQTL SNP. The MARK2 probe cg21609804 contains a miRNA seed region at the interrogated loci.

**Fig. 2:**
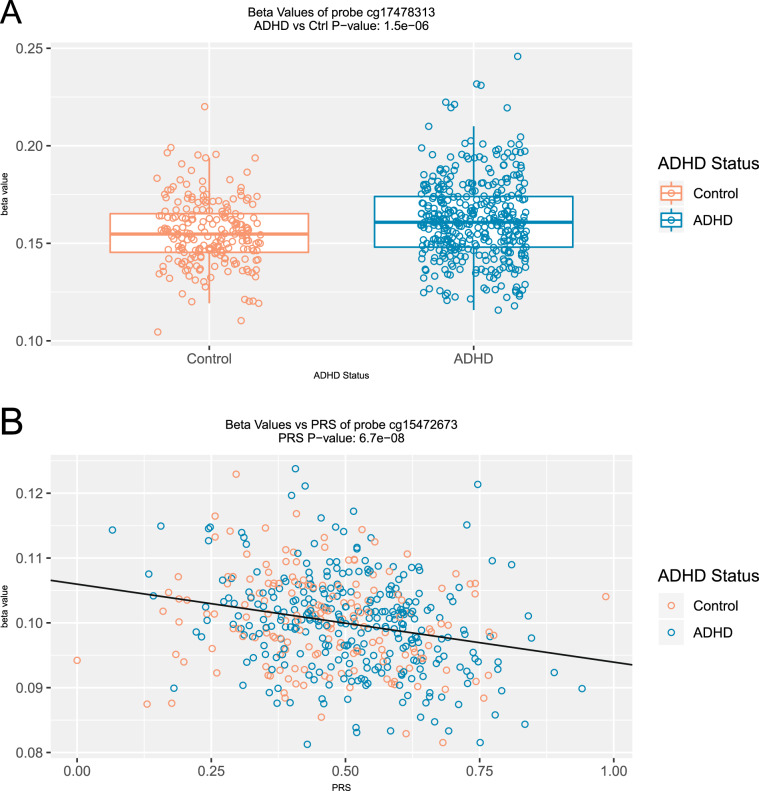
Top-ranked ADHD- and ADHD PRS-associated DMPs. DNA methylation values (beta values) for the top-ranked ADHD- and ADHD PRS-associated probes.

No DMPs passed EWAS significance (*p* = 8.8e–8), which corrects for all 568,281 probes tested. Table 2 shows the 7 DMPs associated with ADHD at our a priori suggestive significance level of *p* < 1e–5. Methylation differences between ADHD cases and controls ranged between 0.3% and 1.4%. The top 100 probes associated with ADHD are provided in Table S5 for descriptive purposes. The top-ranked probe, cg17478313 (*p* = 1.54e–6), shows higher DNA methylation in ADHD cases compared with controls (Δ*β* = 0.93%) and is located in the promotor region of *SLC7A8* (Figs. 1a and 2a). Probe cg21609804 (*p* = 2.82e–6), also with higher methylation (Δ*β* = 1.38%) in ADHD cases, is located in the 3′-UTR of *MARK2* (Fig. 1b). Additional suggestive DMPs were annotated to *PDLIM5*, *VPS28*, *ZNF706*, and *FAM59A*.

**Table 2:** Differentially methylated positions associated with ADHD and the ADHD PRS.

Probe	Coefficient	*P*-value	Chrom	Position	Strand	Gene
*(A) ADHD vs. control*						
cg17478313	0.009285	1.54E–06	14	23653041	−	SLC7A8
cg21609804	0.013772	2.82E–06	11	63678193	+	MARK2
cg03416665	−0.00682	4.64E–06	4	95373403	−	PDLIM5
cg27034450	0.007208	4.82E–06	8	145649435	−	VPS28
cg02466711	−0.00264	6.03E–06	8	102218365	+	ZNF706
cg00964221	−0.00459	8.40E–06	8	30240029	−	Intergenic
cg06972911	−0.00646	9.50E–06	18	30051803	+	FAM59A
*(B) ADHD polygenic risk score*						
cg15472673	−0.01258	6.71E–08	21	34915098	−	GART, SON
cg05348870	0.022926	1.85E–06	19	6671045	+	TNFSF14
cg03391479	0.015117	1.88E–06	4	114901073	+	ARSJ
cg03838680	0.012572	2.22E–06	3	46887195	−	Intergenic
cg00428296	0.015183	2.96E–06	1	230513970	−	PGBD5
cg17233422	0.02449	3.89E–06	3	66467994	+	LRIG1
cg13011002	0.009106	4.48E–06	2	28837591	−	PLB1
cg04453792	0.025432	4.68E–06	16	23158863	−	USP31
cg12237140	0.012843	4.93E–06	19	47988486	−	KPTN
cg16536664	−0.01169	5.61E–06	3	186288091	+	DNAJB11, TBCCD1
cg11425280	−0.01202	5.98E–06	12	104851172	+	CHST11
cg26223996	0.011179	7.34E–06	15	74906395	−	CLK3
cg25332391	0.012247	9.92E–06	5	31505476	+	DROSHA
*(C) ADHD-by-sex interactions*						
cg25779690	−0.01095	1.10E–06	12	56385212	−	RAB5B
cg08698885	−0.00938	1.87E–06	2	96767035	+	Intergenic
cg26975193	−0.00619	2.62E–06	7	158824399	−	VIPR2
cg11411509	−0.01062	3.02E–06	6	33157746	+	COL11A2
cg03537872	−0.02137	3.72E–06	12	109459024	+	SVOP
cg05457620	−0.01166	4.21E–06	5	168310308	+	SLIT3
cg15718572	−0.03317	5.34E–06	1	207818003	+	CR1L
cg23824987	0.00986	8.91E–06	16	69497556	−	CYB5B
cg18031661	0.01126	9.87E–06	5	163133751	+	Intergenic
*(D) ADHD PRS-by-sex interactions*						
cg20954180	0.03722	2.09E–06	3	54606265	+	CACNA2D3
cg06174989	0.07740	2.40E–06	16	90114523	−	LOC100130015
cg08911728	−0.03989	2.89E–06	5	109189484	+	MAN2A1
cg22225943	0.07494	4.55E–06	11	2162536	−	IGF2; INS-IGF2; IGF2AS
cg05407555	−0.01942	9.23E–06	14	106320510	−	Intergenic

All differentially methylated probes with *p*-value ≤ 1e–5 for all models: (A) DMPs associated with ADHD diagnosis, (B) DMPs associated with ADHD polygenic risk (PRS), (C) DMPs showing a sex-by-ADHD interaction, and (D) DMPs showing a sex-by-PRS interaction

### DNA methylation associated with ADHD polygenic risk

We have previously shown that the ADHD PRS is associated with ADHD status (explaining ~4.5% of the variance) in this sample^4^. Here we use the ADHD PRS to investigate the relationship between overall ADHD genetic burden and DNA methylation (Eq. (3)). Again, EWAS results for the PRS are well-controlled (*λ* = 1.08). A Q–Q plot and Manhattan plot are presented in Fig. S6. One probe met our genome-wide significance threshold, cg15472673 (*p* = 6.71E–8), characterized by reduced DNA methylation with higher PRS (Fig. 2b). The association remains (*p* = 9.76e–8), when including ADHD status in the regression model, indicating that the effect is not driven by elevated polygenic burden in ADHD cases. This probe is located in a CpG island of a bivariate promoter between the *GART* and *SON* genes. None of the SNPs included in the PRS are direct mQTLs (see below) for cg15472673 (all mQTL *p*-values > 1e–5), indicating that the association with the PRS is not driven by a simple genetic effect on DNA methylation. DNA methylation levels at 12 other probes were associated with the PRS at *p* < 1.0e–5, 10 of which showed increased methylation with higher PRS (Table 2b). A summary of all findings for ADHD diagnosis and the ADHD PRS is presented in Fig. S7.

### Sex-specific variation in DNA methylation

Because we previously reported an association between ADHD and DNA methylation at sites annotated to *VIPR2* and *MYT1L* specifically in boys^31^, we first examined sex-by-diagnosis interaction effects among all probes annotated to these two genes (239 probes) at an adjusted significance threshold of *p* < 0.0002 (0.05/239). The two strongest sex-by-diagnosis interactions annotated to VIPR2 were for cg26975193 and cg20998127 (sex-by-diagnosis interaction *p* = 7.51e–6 and *p* = 0.000459). Supporting the finding from our previous study, males with ADHD had lower methylation compared with male controls at both sites (Δ*β* = −0.22%, *p* = 0.0185; Δ*β* = −0.51%, *p* = 3.99e–5). However, among females, ADHD cases had higher methylation levels than controls at cg26975193 (Δ*β* = 0.35%, *p* = 0.00597) and were not significantly different from controls at cg20998127 (Δ*β* = 0.14%, *p* = 0.384). To ensure replication of the hypomethylation effect in boys with ADHD reported by Wilmot et al.^31^, we removed the samples included in that previous report (*n* = 73 that survived QC for the current probe set). Effects among males were consistent at both sites (for cg26975193: Δ*β* = −0.19%, *p* = 0.0819; for cg20998127: Δ*β* = −0.51%, *p* = 4.14e–4).

For *MYT1L*, our previously reported finding did not survive multiple-testing correction (minimum sex-by-diagnosis interaction *p*-value = 0.0039 for cg02870147; males Δ*β* = −0.49%), although the direction of effect at this site (lower methylation among males with ADHD) was consistent with our previous data, even with prior samples (*n* = 73) removed (males Δ*β* = −0.42%). Likewise, no probes annotated to *MYT1L* showed main effects that passed the Bonferroni threshold (minimum *p*-value of 0.00395 for cg22140907; Δ*β* = −0.31%). All results from the analysis of these genes are reported in Table S6.

We next performed an EWAS of sex-specific DMPs associated with both ADHD and the ADHD PRS. No sex interactions were significant at the EWAS-wide threshold, although nine probes show sex-by-diagnosis interactions (Table 2c) and five probes show sex-by-PRS interactions (Table 2d) at our suggestive significance threshold (*p* < 1e–5).

### Differentially methylated regions

The results from all DMP analyses were used to investigate differentially methylated regions (DMRs) using the Comb-p software tool^41^. A single significant DMR on chromosome 6 was identified (Šidák-corrected *p* = 3.4e–5), which contained eight probes associated with the ADHD PRS in a sex-specific manner (Fig. S8). Specifically, among females a higher PRS was associated with higher methylation levels, and an opposite (though much weaker) relationship was seen among males. This DMR (chr6: 31148383–31148553) lies within the major histocompatibility complex, ~3 kb upstream of *PSORS1C3*.

### DNA methylation quantitative trait loci

**Fig. 3:**
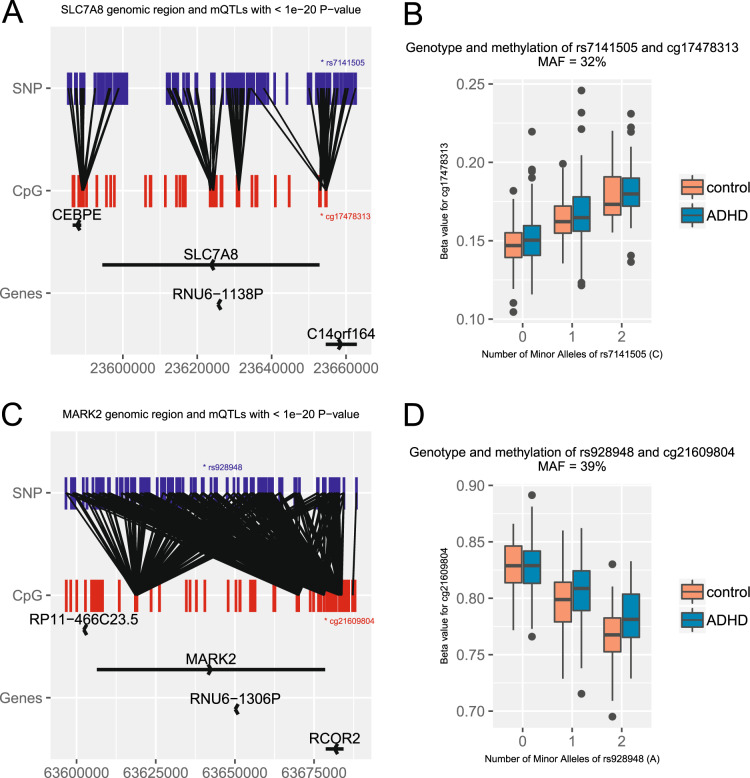
DNA Methylation QTLs within SLC7A8 and MARK2. The genomic region around SLC7A8 (**a**), showing locations of cg17478313 and the most significant mQTL rs7141505. Similarly, the genomic region around MARK2 (**c**) with locations of cg21609804 and the most significant mQTL rs928948. For clarity, only mQTLs with *p* < 1e–20 in each region are shown. The methylation values (beta values) for both cg17478313 and cg21609804, stratified by mQTL genotype, are also shown (**b**, **d**).

We identified methylation quantitative trait loci (mQTL) associated with all DMPs suggestively associated with (a) ADHD or (b) the ADHD PRS (Table 2). DNA methylation at the two top-ranked ADHD-associated DMPs (cg17478313 annotated to *SLC7A8* and cg21609804 annotated to *MARK2*, Table 2a) was significantly associated with genotypes at nearby SNPs (Fig. 3). The *SLC7A8* probe (cg17478313) was associated with SNP rs7141505 (*p* = 1.2e–46), located in the gene’s promoter region. The *MARK2* probe (cg21609804) was associated with an intronic SNP rs928948 (38 kb upstream of the CpG) at *p* = 2.07e–59 (Fig. 1b). Furthermore, for both mQTLs, the relationship between genotype and DNA methylation level is similar in both ADHD cases and controls (Fig. 3b, d). Also, for both cg17478313 and cg21609804, both ADHD status (*p* = 2.2e–4 and *p* = 1.85e–4) and genotype (*p* = 4.4e–45 and *p* = 8.9e–58) are associated with methylation levels when included together in the regression models. These results indicate that the mQTL effects are not simply due to allele frequency differences between ADHD cases and controls. An additional 279 SNPs are associated with methylation levels at these two DMPs (31 for cg17478313 and 248 for cg21609804), and pass our experiment-wide mQTL significance threshold (*p* < 1.8e–10).

For the ADHD PRS, of all the associated DMPs (at *p* < 1e–5), significant mQTLs were found only for cg04453792 annotated to *USP31* (*p* < 1.8e–10). All mQTL analysis results are provided in Table S7.

To determine whether mQTL variants associated with the top-ranked DMPs (for both ADHD and the ADHD PRS) were also implicated in GWAS for ADHD (i.e., pleiotropic for methylation and ADHD), we examined the results from the PGC ADHD GWAS meta-analysis^3^ in the regions of *SLC7A8*, *MARK2*, *GART*, *SON*, *USP31*, and *LOC100130015* (Table 2 and Table S7). We assessed all GWAS SNPs included in our mQTL analysis that were in linkage disequilibrium with these six genes or their putative regulatory regions (20 kb upstream or downstream of each gene, see Supplementary Materials). No genome-wide significant (or suggestive) ADHD-associated SNPs assigned to MARK2 (minimum *p* = 0.02), SLC7A8 (minimum *p* = 0.3), GART/SON (minimum *p* = 0.029), USP31 (minimum *p* = 0.069), or LOC100130015 (minimum *p* = 0.011) were seen in the GWAS meta-analysis, indicating there is no evidence of pleiotropy for these mQTLs.

To expand the investigation of pleiotropy, all mQTLs observed in our cohort (not just those associated with DMPs) were tested for colocalization with variants associated with ADHD in the recent ADHD GWAS meta-analysis conducted by the PGC^3^. Of the 12 ADHD-associated regions identified in the GWAS meta-analysis, 11 contained genome-wide significant *cis*-mQTLs in our cohort (Table S3). Evidence for colocalization/pleiotropy was found for variants in 5 of the 12 ADHD-associated regions (Table S4). For two of the regions, 12q21.33 and 15q21.1 (Figs. S9 and S10), both the SMR and *coloc* methods identify the same causal SNPs (rs2279574, SMR *p* = 2.3e–8, *coloc* posterior probability = 0.98, and rs1656622, SMR *p* = 3.6e–5, *coloc* posterior probability = 0.82). SNP rs2279574 is a missense variant within *DUSP6*, and rs1656622 lies within an intron of *SEMA6D*. All significant mQTLs in the 12 ADHD-associated regions are included in Table S10.

### Gene set enrichment with DM probes

For exploratory and hypothesis-generating purposes, we report gene set findings. DMPs nominally associated with the ADHD PRS (*p* < 0.001, the recommended default in the *methylGSA* package)^49^ are annotated to 91 genes with elevated expression (five-fold higher-than-average expression in all other tissues, see Methods) in the brain (unadjusted enrichment *p*-value = 0.0097). Several (Fig. S11) relate to ion channels (e.g., *KCNIP1*, *KCNK10*, *CACNA1E*, and *CACNB4*) or are involved in cell adhesion (e.g., *NCAM2*, *NRXN1*, *CNTNAP2*, and *CDH22*), both previously implicated in ADHD or other psychiatric traits^54–58^. No GO categories were significantly enriched with either ADHD-associated or ADHD PRS-associated DMPs after multiple testing correction. All enrichment analysis results are shown in Table S8.

## Discussion

This is the first large EWAS to examine well-characterized ADHD cases and controls, including more ADHD cases than any prior study of DNA methylation in ADHD. It is distinguished by the inclusion of polygenic risk effects—not previously studied in ADHD in an EWAS context—and the examination of sex effects. Furthermore, the inclusion of genetic effects, crucial for interpretation, has rarely been done in ADHD methylation studies. The findings make contributions pertaining to potential peripheral biomarkers for ADHD and ADHD genetic risk, provide additional evidence for the genetic regulation of many disease-associated methylation differences in ADHD, and suggest regulation of DNA methylation as a plausible mechanism for ADHD risk variants identified in GWAS. The study identifies suggestive new candidates to pursue (e.g., *VIPR2*, *MARK2*, *SLC7A8*, *SON*, and *PSORS1C3*), and provides new data for considering replications, or lack thereof, with previous epidemiological studies.

The study’s primary limitations are that even with our respectable sample size compared with the literature in this area, the sample is, like prior studies of DNA methylation in ADHD, probably underpowered to identify small effects. Our sample was well powered (80%) to detect methylation differences of 1% between cases and controls at ~68% of sites (Fig. S8); however, many sites show even smaller effects in our dataset and in others^33,52^. Thus, these findings should be viewed in a discovery context awaiting replication and as a guide for planning future studies. Furthermore, given the cross-sectional nature of this study, it is not possible to examine causality. As mentioned in the introduction, it is possible that the observed methylation differences are not causal of, but are caused by, ADHD disease processes.

With these strengths and limitations in mind, five main findings are noteworthy. First, our previous report of lower DNA methylation in *VIPR2* in never-medicated boys^31^ was supported in a targeted analysis after removing children used in our prior report, although at a smaller effect size, suggesting that this sex-specific effect is reproducible. Sites annotated to *VIPR2* have also been identified as differentially methylated in two other studies of ADHD^28,32^, but with effects varying in direction. Given that we observe a sex-specific effect in *VIPR2* (hypomethylation in male cases, and hypermethylation in female cases) and other studies did not stratify by sex, it is possible that the inconsistencies across studies related to the direction of effect are due to differences in the sex ratio of the samples studied. Other differences across study samples, such as differences in age or specific environmental exposures, may also contribute to the inconsistent direction of effect. The role of *VIPR2*, a receptor for a small neuropeptide implicated in a wide range of biological functions, needs more exploration. Genetic variation in *VIPR2* has been associated with schizophrenia^59,60^, and in one candidate gene study, with mood disorders^61^.

Second, we identified suggestive findings for a small number of DNA methylation sites that can provide possible biomarkers for ADHD and associated phenotypes. We note that the top two DMPs are both located in regulatory regions of their respective genes (*SLC7A8* and *MARK2*). *SLC7A8* is a sodium-independent solute transporter of neutral amino acids^62^ and plays a role in metal ion homeostasis and toxicity^63^ including mercury toxicity^64^. The DMP annotated to *MARK2* is at the seed of two miRNAs, hsa-miR-1199-5p, and hsa-miR6751-3P, suggesting that differential methylation at this locus may indirectly affect gene expression through regulation of miRNAs. The full list of miRNA targets for this sequence using miRWalk^47^ is provided at https://github.com/pryabinin/ohsu_adhd_ewas. *MARK2* is a regulator of cell polarity and microtubule dynamics^65,66^, is required for neurite outgrowth^67^, and has recently been implicated in both Alzheimer’s disease and bipolar disorder^68^.

Both of the top-ranked DMPs are also associated with nearby SNPs, indicating the complexity of the association between DNA methylation in these genes and ADHD. Of note, a variant in *MARK2* has previously been associated with blood pressure response to methylphenidate^69^. This SNP, rs12099085 (27 kb downstream of the top-ranked mQTL), is also an mQTL suggestively associated with methylation at sites within *MARK2* (minimum mQTL *p* = 3.8e–8), indicating that the variant might act through the regulation of DNA methylation.

Third, we identified one DMP significantly associated with polygenic risk for ADHD (ADHD PRS), and another 12 DMPs suggestively associated. The strongest association was at a site in the bivariate promoter for *GART* and *SON*, with higher PRS associated with reduced methylation. *SON* SNPs have been implicated in a GWAS for educational attainment^70^, which is noteworthy given the known relationship between ADHD and academic underachievement^71^, as well as the evidence for a shared genetic basis for ADHD and educational attainment^72^. De novo mutations in *SON* have been linked to intellectual disability, and gene expression studies suggest that it is a master regulator of genes involved in neurodevelopment^73^.

Fourth, we found a single DMR, near *PSORS1C3*, significantly associated with the ADHD PRS in a sex-specific manner. A previous study found that a DMR in this same region was hypomethylated in major depressive disorder (MDD) suicide cases^74^, although it did not assess sex-specific effects.

Finally, we found significant evidence of colocalization for mQTLs observed in our cohort and ADHD-associated SNPs, suggesting that regulation of DNA methylation may be a mechanism by which many ADHD risk variants operate.

The small number of DMPs and DMRs we observe, and the small effect sizes, are consistent with a recent EWAS meta-analysis of ADHD symptoms in adults^33^. That study of three population-based cohorts found three suggestively associated DMPs, none of which overlap with the DMPs reported here. The lack of overlap is not surprising, however, given the amount of heterogeneity across cohorts reported in the meta-analysis. That study also failed to see an overlap between ADHD-related DMPs and ADHD GWAS hits from the PGC. However, consistent with our findings, the study found *cis*-mQTLs for their top-ranked DMP, highlighting the importance of genetic effects.

In conclusion, our findings suggest that further study of DNA methylation in ADHD can be productive to identify biomarkers of illness, and potentially, disease mechanisms, although ultimately multi-position algorithms will likely be necessary to create clinically useful biomarkers^10^. It also appears likely to be helpful in mapping potential causal routes for genetic influences on ADHD. Future work will examine sex and medication effects, moderators of change and/or stability of DNA methylation over time, map backward to environmental correlates, and evaluate the extent to which identified genes or systems show methylation effects earlier or later in development.

## Supplementary information

Manuscript Supplement

Supplemental Table S5

Supplemental Table S6

Supplemental Table S7

Supplemental Table S8

Supplemental Table S9

Supplemental Table S10
