# Supplementary material for: Large epigenome-wide association study of childhood ADHD identifies peripheral DNA methylation associated with disease and polygenic risk burden
Source: Transl Psychiatry. 2020 Jan 21;10:8. doi: 10.1038/s41398-020-0710-4 (PMC7026179; doi:10.1038/s41398-020-0710-4)
Supplement: Supplementary file 1 — Manuscript Supplement [file 41398_2020_710_MOESM1_ESM.docx]

Supplementary Materials

**Additional Methods: Pages 1 - 4**

**References: Page 5**

**Tables and Figures: Pages 7 - 20**

**Case Identification**

All families underwent a multi-gate screening process to establish eligibility and diagnostic group assignment that included a parent-clinician clinical structured diagnostic parent interview (Kiddie Schedule for Affective Disorders and Schizophrenia for School-Age Children—Epidemiologic Version [KSAD-S-E]) [1], parent and teacher standardized rating forms that assessed symptoms and impairment, clinician observations, and child completion of a short form of the Wechsler Intelligence Scales for Children-Fourth Edition [2]. A clinical diagnostic team (a board-certified child psychiatrist and licensed child neuropsychologist) then reviewed all information (blind to all epigenetic data) in order to implement a best estimate diagnostic procedure. Their agreement rate was acceptable for ADHD (*k* >0.80; all subtypes were allowed) and for all disorders with base-rate >5% in the study (*k* >0.75). They resolved disagreements by consensus discussion. Exclusion criteria include an estimated Full Scale IQ < 75; diagnosis of current major depressive episode, lifetime mania or psychosis, pervasive developmental disorder (including autism), learning disability, or major medical/neurological disorders or injuries.

**Sample QC and Filtering**

The Illumina BeadArray Controls Reporter was run using the default parameters and all samples that it flagged were removed. The beta distributions and total CpG intensity distribution of each sample was manually inspected, and samples removed if the distributions were determined to be abnormal in comparison with the rest of the cohort.

Samples were clustered by multidimensional scaling using 635,994 CpGs which have > 0.1 coefficient of variance in order to identify outlier samples as well as confirm sex annotations.

Two samples which were annotated as males were clustering with the females and were removed from further analysis. Data was normalized using various methods from the lumi [3] and wateRmelon [4] packages in R and compared against each other by the resulting beta and intensity distributions as well as three wateRmelon QC statistics (using imprinted regions, SNP QC probes, and X-inactivation). The data was adjusted for background and color bias, and smooth quantile normalization was applied using the lumi package.

The methylation values of the SNP QC probes included on the chip were compared to the genotypes. Four samples had a correlation between methylation and genotype less than 0.8 and were removed from further analysis.

Eleven samples were removed from the analysis for sub-threshold ADHD diagnosis (e.g. 5 symptoms), and 8 samples were removed because they were diagnosed with the hyperactive ADHD subtype. Intensity values were plotted to confirm that the cleaning steps so far have removed low quality samples (Figure S2).

**Age Prediction**

The reported ages for all study participants were compared to predicted ages using cleaned, normalized methylation data for probes previously associated with age [5] (Figure S3).

**Probe Filtering**

Probes were removed for the following reasons: detection p-value < 0.01 in at least one sample, multi-mapping probes, probes which had an underlying SNP with a MAF of >= 0.01 in any examined subpopulation [6] or according to the Illumina manifest, and non-autosomal loci (Table S2). Outlier values were identified as values >4 times the interquartile range away from the mean for each probe, and were subsequently flagged as missing for further analysis.

**Cell Type Correction**

Reference-free cell type prediction was performed using RefFreeEWAS [7]. Briefly, the package uses non-negative matrix factorization to predict the cell type profiles and cell type proportions present in a data set. To speed these computations, only the top 20,000 most variable probes (by standard deviation) were used. Models for 1 through 10 different cell types were calculated using 25 iterations for each model. The optimal model to use was calculated using 900 bootstraps for each model with 5 iterations per bootstrap and the model which minimized the median of the deviances of the bootstraps was chosen as the optimal model. The predicted cell type proportions from the 10 cell type model was used to adjust the beta values in order to account for cell type differences between samples by applying a linear model to predict the beta values from the centered (at zero) cell type proportions in each sample:

$$Beta value \approx\alpha+ \beta*centered cell proportions$$

The product of the beta coefficients (of the centered cell proportions) and the centered cell proportions was subtracted from the beta values in order to calculate the cell type adjusted beta values:

$$Adjusted Beta Value \approx Beta Value- \hat{\beta}*centered cell proportions$$

**Outlier Sample Removal**

Outlier sample detection was performed by calculating the number of probes that differed greatly from many other unrelated samples. For each sample the absolute difference across all probes was calculated relative to a random sampling of 100 unrelated samples. The median number of probes differing in each pairing was calculated for each sample. For one sample, the median count of CpG differences >0.4 was 8,317—far more than any other sample (the next largest median count was 408). This sample was removed from further analysis, resulting in a final sample of N=604.

**Polygenic Risk Score Calculation**

The polygenic risk score (PGS) was constructed using the European-ancestry PGC meta-analysis [8] as the discovery data set (19099 ADHD cases, 34194 controls). Genotypes for our cohort were imputed using 1000 genomes (1KG phase 3) as the reference panel (https://mathgen.stats.ox.ac.uk/impute/1000GP_Phase3.html). Only SNPs with INFO score (imputation quality) > 0.8 in both the PGC meta-analysis and our data were considered. SNPs were clumped based on r2 < 0.1 using genotypes from European samples of the 1KG phase 3 using PLINK v1.9 (https://www.cog-genomics.org/plink2). From this filtered (LD clumped) SNP list, SNPs with p < 0.5 were selected for the polygenic score computation (N=139934). The PGS was calculated for all subjects in the target data set by multiplying the number of risk alleles (0, 1, or 2) by the log(odds ratio) of that SNP in the discovery data set and averaging over all SNPs. The allelic scoring function (--score) in PLINK v1.9 was used for the PGS calculation. Methods and scripts for genomic analyses are found at <https://github.com/pryabinin/ohsu_adhd_gwas>.

**DNA Methylation Quantitative Trait Loci (mQTL) Calculation**

mQTLs between all methylation probes and SNPs on the same chromosome were calculated using the GEM [9] package (189,872,556,295 total tests performed), and the mQTLs with a raw p-value of 1e-13 are reported in the github repository. mQTLs were calculated using an additive linear model to identify a relationship between methylation beta value and the number of minor alleles of each SNP, with covariates for age, sex and three genomic principal components. To produce figures 2A and 2C, the chromosome-wide mQTL results were subset to mQTLs lying within each region and with p-value < 1e-20.

**Annotation of SNPs in the Region of mQTLs Associated with Top-ranked DMPs**

A LD region around the genes of interest (MARK2, SLC7A8, GART, SON, USP31, and LOC100130015) was calculated by using genotype data from European (CEU) samples from the 1000 genomes project. For each gene, the LD blocks were calculated for all SNPs within 100kb of the start and stop transcription sites using the “Confidence Intervals” algorithm [10] as implemented in Haploview (version 4.2). SNPs within 20kb of the gene’s transcription start and stop sites were automatically included within the LD region of the gene. Additionally, any other SNP that shared a LD block with an automatically included SNP was also added to the LD region.

**Functional Significance**

Downstream interpretation of associated DNA methylation probes was performed using *Genetica*, a custom R package developed in our lab (<https://github.com/bhattp09/genetica>). *Genetica* aims to provide an overall review of existing knowledge for a list of genes/SNPs/probes of interest. *Genetica* was used to summarize publicly-available information for the top-ranked genes, SNPs and probes from our DMP and mQTL analyses. The databases and datasets queried were: GTEx (<https://gtexportal.org/home/>), Gene Ontology (<http://geneontology.org/>), GWAS Catalog (<https://www.ebi.ac.uk/gwas/>), and mQTLs in the AIRES study [11], Fetal Brain [12] and Blood methylome [13], as well as our own dataset (see above).

Gene Cards (Wiezman Institute of Science, <http://www.genecards.org/>) was used to identify enhancers that have been associated with genes of interest.

*Genetica* outputs the Ensembl ID, chromosome and position of each gene, or annotated gene for SNPs and methylation probes, in both hg19 and hg38 builds, if available.

*Genetica* queries the NHGRI-EBI GWAS Catalog [14] to determine if a gene (or annotated gene for a SNP or methylation probe query) has been previously identified as a top result in a published genome-wide association study. The output includes the publication’s reference along with the associated phenotype.

**Statistical Power**

The statistical power to detect an effect size of 1% difference in DNA methylation between cases and controls at p-value < 8.8e-8 was calculated for all probes included in our analysis. Power calculations were done using each probe’s variance across all subjects, and the number for cases and controls in the experiment. The proportion of probes that achieved a particular power threshold was calculated for a range of powers from 0 to 1 (see Supplementary Figure S8).

**References**

1. Puig-Antich J, Ryan N. Kiddie schedule for affective disorders and schizophrenia. Pittsburgh, PA: Western Psychiatric Institute; 1986.

2. Wechsler D. The Wechsler Intelligence Scale for Children: Administration and Scoring Manual. the Psychological Corporation. San Antonio, TX. 2003;

3. Du P, Kibbe WA, Lin SM. lumi: a pipeline for processing Illumina microarray. Bioinformatics. 2008;24:1547–8.

4. Pidsley R, Y Wong CC, Volta M, Lunnon K, Mill J, Schalkwyk LC. A data-driven approach to preprocessing Illumina 450K methylation array data. BMC Genomics. 2013;14:293.

5. Horvath S. DNA methylation age of human tissues and cell types. Genome Biology. 2013;14:R115.

6. McCartney DL, Walker RM, Morris SW, McIntosh AM, Porteous DJ, Evans KL. Identification of polymorphic and off-target probe binding sites on the Illumina Infinium MethylationEPIC BeadChip. Genomics Data. 2016;9:22–4.

7. Houseman EA, Kile ML, Christiani DC, Ince TA, Kelsey KT, Marsit CJ. Reference-free deconvolution of DNA methylation data and mediation by cell composition effects. BMC Bioinformatics [Internet]. 2016 [cited 2018 May 1];17. Available from: http://bmcbioinformatics.biomedcentral.com/articles/10.1186/s12859-016-1140-4

8. Demontis D, Walters RK, Martin J, Mattheisen M, Als TD, Agerbo E, et al. Discovery of the first genome-wide significant risk loci for attention deficit/hyperactivity disorder. Nature Genetics. 2019;51:63.

9. Pan H, Holbrook JD, Karnani N, Kwoh CK. Gene, Environment and Methylation (GEM): a tool suite to efficiently navigate large scale epigenome wide association studies and integrate genotype and interaction between genotype and environment. BMC Bioinformatics. 2016;17:299.

10. Gabriel SB, Schaffner SF, Nguyen H, Moore JM, Roy J, Blumenstiel B, et al. The Structure of Haplotype Blocks in the Human Genome. Science. 2002;296:2225–9.

11. Relton CL, Gaunt T, McArdle W, Ho K, Duggirala A, Shihab H, et al. Data Resource Profile: Accessible Resource for Integrated Epigenomic Studies (ARIES). Int J Epidemiol. 2015;44:1181–90.

12. Spiers H, Hannon E, Schalkwyk LC, Smith R, Wong CCY, O’Donovan MC, et al. Methylomic trajectories across human fetal brain development. Genome Res. 2015;gr.180273.114.

13. Hannon E, Dempster E, Viana J, Burrage J, Smith AR, Macdonald R, et al. An integrated genetic-epigenetic analysis of schizophrenia: evidence for co-localization of genetic associations and differential DNA methylation. Genome Biology. 2016;17:176.

14. Buniello A, MacArthur JAL, Cerezo M, Harris LW, Hayhurst J, Malangone C, et al. The NHGRI-EBI GWAS Catalog of published genome-wide association studies, targeted arrays and summary statistics 2019. Nucleic Acids Res. 2019;47:D1005–12.

Tables:

Table S1. Medication Frequencies

| **Preparation** | **% of participants with lifetime medication use** | N  (out of 180) | **% of ADHD participants** | N  (out of 391) |
| --- | --- | --- | --- | --- |
| Adderall or Adderall X (mixed amphetamine/dextroamphetamine) | 17.8% | 32 | 8.2% | 32 |
| Vyvanse (lisdexamfetamine dimesylate) | 5.6% | 10 | 2.6% | 10 |
| Dexedrine (Dextroamphetamine) | 1.7% | 3 | 0.8% | 3 |
| **Total amphetamine salts** | **25.0%** | **45** | **11.5%** | **45** |
|  |  |  |  |  |
| Ritalin or Ritalin LA (methylphenidate) | 9.4% | 17 | 4.3% | 17 |
| Focalin (dexmethylphenidate) | 3.9% | 7 | 1.8% | 7 |
| Quillavent (methylphenidate) | 0.6% | 1 | 0.3% | 1 |
| Concerta (methylphenidate) | 27.2% | 49 | 12.5% | 49 |
| Daytrana (methylphenidate) | 1.7% | 3 | 0.8% | 3 |
| Metadate (methylphenidate) | 3.3% | 6 | 1.5% | 6 |
| Methylin (methylphenidate) | 1.7% | 3 | 0.8% | 3 |
| **Total methylphenidate preparations** | **47.8%** | **86** | **22.0%** | **86** |
|  |  |  |  |  |
| **Unknown stimulant** | **10.6%** | **19** | **4.9%** | **19** |
|  |  |  |  |  |
| **No medication use** | n/a | n/a | **54.5%** | **213** |
| Multiple stimulant medications only | 10.6% | 19 | 4.9% | 19 |
| Multiple stimulant and psych medications *(Celexa, Clonidine, Zoloft, Prozac, Buspar, Remeron, Trazadone, Klonopin, Guanfacine, Strattera)* | 3.9% | 7 | 1.8% | 7 |
| Ativan only | 0.6% | 1 | 0.0% | 0 |
| Depakote only | 0.6% | 1 | 0.0% | 0 |
| Strattera only | 0.6% | 1 | 0.3% | 1 |
| Valium only | 0.6% | 1 | 0.3% | 1 |
| **Total psych meds** | **16.7%** | **30** | **7.2%** | **28** |
|  | 100.0% | 180.00 | 100.0% | 391.00 |

Note: These values reflect the percentages for the complete N=604 sample.

Table S2. Probe Filtering

| **Reason for Removal** | **# Probes Removed** | **# Probes Remaining** | **% Probes Remaining** |
| --- | --- | --- | --- |
|  | 0 | 866,895 | 100 |
| Low Detection Rate | 49,622 | 817,273 | 94.3 |
| Multimapping probe | 42,273 | 775,000 | 89.4 |
| SNP QC Probe | 59 | 774,941 | 89.4 |
| SNP underlying probe | 192,401 | 582,540 | 74.8 |
| Non-autosomal probe | 14,259 | **568,281** | 73.2 |

Table S3. Summary of mQTLs in ADHD-associated Regions from the PGC GWAS Meta-analysis

| Region | mQTLs p < 1.8e-10 |
| --- | --- |
| chr1: 43.9 – 44.4Mb | 941 SNPs, 38 probes |
| chr1: 96.3 – 96.8Mb | 528 SNPs, 3 probes |
| chr2: 214.9 – 215.4Mb | 52 SNPs, 2 probes |
| chr3: 20.4 – 20.9Mb | -- |
| chr4: 30.9 – 31.4Mb | 193 SNPs, 3 probes |
| chr5: 87.6 – 88.1Mb | 99 SNPs, 5 probes |
| chr7: 113.8 – 114.3Mb | 240 SNPs, 3 probes |
| chr8: 34.1 – 34.6Mb | 1122 SNPs, 2 probe |
| chr10: 106.5 – 107Mb | 470 SNPs, 3 probes |
| chr12: 89.5 – 90Mb | 540 SNPs, 19 probes |
| chr15: 47.5 – 48Mb | 797 SNPs, 10 probes |
| chr16: 72.3 – 72.8Mb | 82 SNPs, 2 probes |

Table S4. Colocalized mQTLs

| Chr | Probe | Probe Pos. | SNP | SNP Pos. | mQTL Pval | GWAS Pval | SMR Pval | HEIDI Pval | coloc PP.H3 | coloc PP.H4 | coloc SNP | coloc SNP  PP.H4 |
| --- | --- | --- | --- | --- | --- | --- | --- | --- | --- | --- | --- | --- |
| 1 | cg02687548 | 44006614 | rs10789434 | 43976621 | 8.85E-10 | 2.02E-07 | **7.38E-05** | **0.1797** | **0.99928433** | 0.0007099 | rs1889588 | 0.14902487 |
| 1 | cg15268136 | 44031309 | rs11210871 | 44029353 | 2.25E-09 | 4.19E-09 | **2.88E-05** | **0.0502** | **0.99592189** | 0.0030869 | rs11210871 | 0.59477625 |
| 1 | cg22875872 | 44031756 | rs674725 | 44061795 | 1.95E-16 | 7.99E-09 | **2.34E-06** | **0.4022** | **0.99767039** | 0.00232957 | rs674725 | 0.21569976 |
| 1 | cg09884599 | 44044046 | rs2819340 | 44039710 | 1.13E-09 | 1.12E-08 | **3.05E-05** | **0.5756** | **0.99706683** | 0.00273321 | rs2004899 | 0.27826699 |
| 1 | cg17737314 | 44114355 | rs11420276 | 44184192 | 6.40E-05 | 2.14E-13 | NA | NA | 0.21717546 | **0.76103744** | rs11420276 | 0.13421026 |
| 1 | cg00946598 | 44117093 | rs517191 | 44116466 | 6.33E-09 | 1.66E-10 | **1.69E-05** | **0.0548** | 0.33769293 | 0.6621123 | rs60879458 | 0.35716398 |
| 1 | cg22889192 | 44154206 | rs61768374 | 44151633 | 6.53E-09 | 1.20E-12 | **6.94E-06** | **0.5179** | 0.03153321 | **0.96843919** | rs17531412 | 0.152925 |
| 1 | cg09989037 | 44300942 | rs803679 | 44349405 | 1.95E-10 | 2.90E-07 | **6.45E-05** | **0.1310** | **0.99333144** | 0.0065678 | rs55803885 | 0.09042623 |
| 1 | cg21138201 | 44313837 | rs12040333 | 44314041 | 4.83E-48 | 1.18E-04 | **2.00E-04** | **0.0710** | **0.99999939** | 5.69E-07 | rs12040333 | 0.9995214 |
| 1 | cg22384188 | 44347398 | rs11210935 | 44369444 | 3.83E-17 | 2.44E-07 | **1.08E-05** | **0.4801** | **0.99982574** | 0.00017422 | rs11210935 | 0.606060532 |
| 5 | cg01089416 | 87677857 | rs1823016 | 87612658 | 2.81E-15 | 5.41E-06 | **8.25E-05** | **0.2955** | 0.72148632 | 0.27746861 | rs7708715 | 0.08345585 |
| 5 | cg07922879 | 87980629 | rs35546989 | 88034867 | 1.52E-07 | 1.19E-07 | NA | NA | 0.16847567 | **0.8294972** | rs35546989 | 0.1486656 |
| 7 | cg13077160 | 114142961 | rs9969232 | 114158954 | 1.11E-05 | 5.60E-08 | NA | NA | 0.11340885 | **0.79440439** | rs9969232 | 0.38005002 |
| 12 | cg16063162 | 89721406 | rs797091 | 89724927 | 1.45E-10 | 5.09E-07 | **7.89E-05** | **0.0982** | 0.61554154 | 0.38387057 | rs797091 | **0.9979347** |
| 12 | cg20143530 | 89747082 | rs704061 | 89771903 | 3.25E-04 | 2.15E-09 | NA | NA | 0.07411316 | **0.81037068** | rs704061 | 0.16113435 |
| 12 | cg08802841 | 89748726 | rs704061 | 89771903 | 8.85E-258 | 2.15E-09 | **4.07E-09** | 0.0159 | 0.01893042 | **0.9810634** | rs2279574 | **1** |
| 12 | cg15296664 | 89748773 | rs2279574 | 89745477 | 4.30E-154 | 4.75E-09 | **1.17E-08** | 0.0294 | 0.01892756 | **0.98106627** | rs2279574 | **0.999751** |
| 12 | cg13519918 | 89748985 | rs2279574 | 89745477 | 3.46E-60 | 4.75E-09 | **3.75E-08** | **0.0799** | 0.01878114 | **0.98121273** | rs2279574 | **0.985534247** |
| 12 | cg22466678 | 89749033 | rs2279574 | 89745477 | 7.31E-83 | 4.75E-09 | **2.25E-08** | **0.0697** | 0.01580164 | **0.98419315** | rs2279574 | 0.687896605 |
| 12 | cg00690181 | 89764656 | rs1427829 | 89760744 | 5.38E-05 | 1.82E-09 | NA | NA | 0.02358226 | **0.94669173** | rs1427829 | 0.20329731 |
| 15 | cg04986098 | 47763972 | rs1656622 | 47813909 | 4.77E-14 | 8.07E-07 | **3.58E-05** | **0.4335** | 0.17905777 | **0.81965151** | rs1656622 | 0.51264031 |

Significant results are in bold. The significance criteria for the SMR method are an SMR p-value < 4e-4 and a HEIDI p-value > 0.05. For the coloc method, a posterior probability of colocalization (PP.H4) > 0.75 is considered significant. The highlighted rows are those variants for which both methods agree there is significant evidence of pleiotropy/causality.

Figure S1. QC and Analysis Workflow Overview

Figure S2. Probe Intensity Values


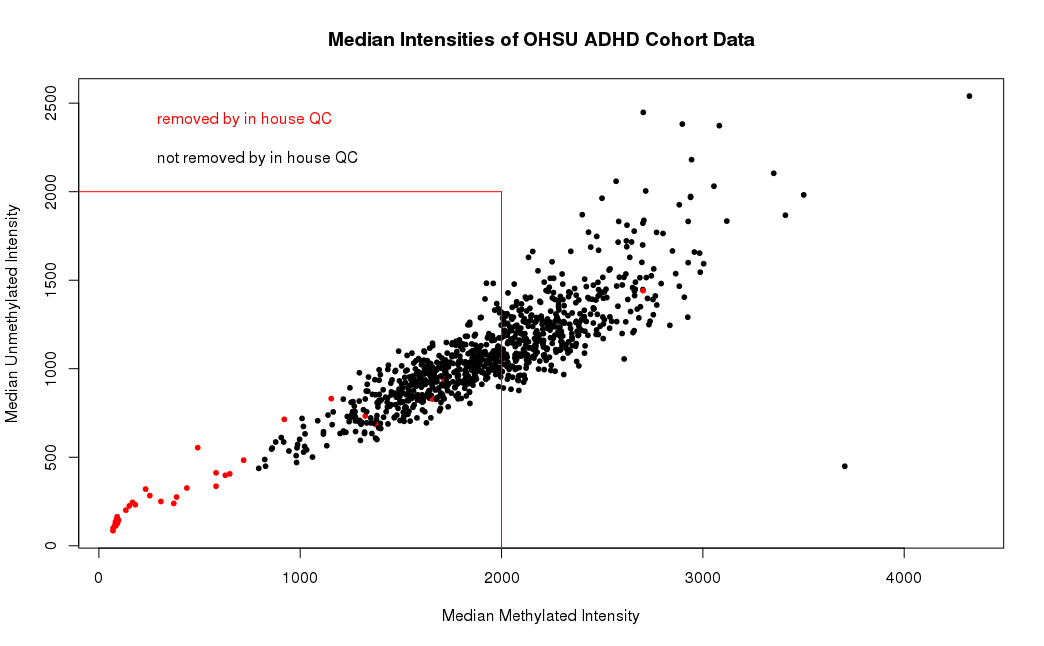


Figure S3: Statistical Power Across All Methylation Probes

The proportion of methylation probes for which the study had a given statistical power to detect a 1% difference in methylation is shown. At approximately 68% percent of sites across the genome, the study had 80% power to detect a 1% difference.

Figure S4. Methylation Age Prediction

The association between reported age and predicted age (based on DNA methylation) did not differ between ADHD cases and controls (p=0.98).

Figure S5. Manhattan and Q-Q Plots for ADHD Diagnosis


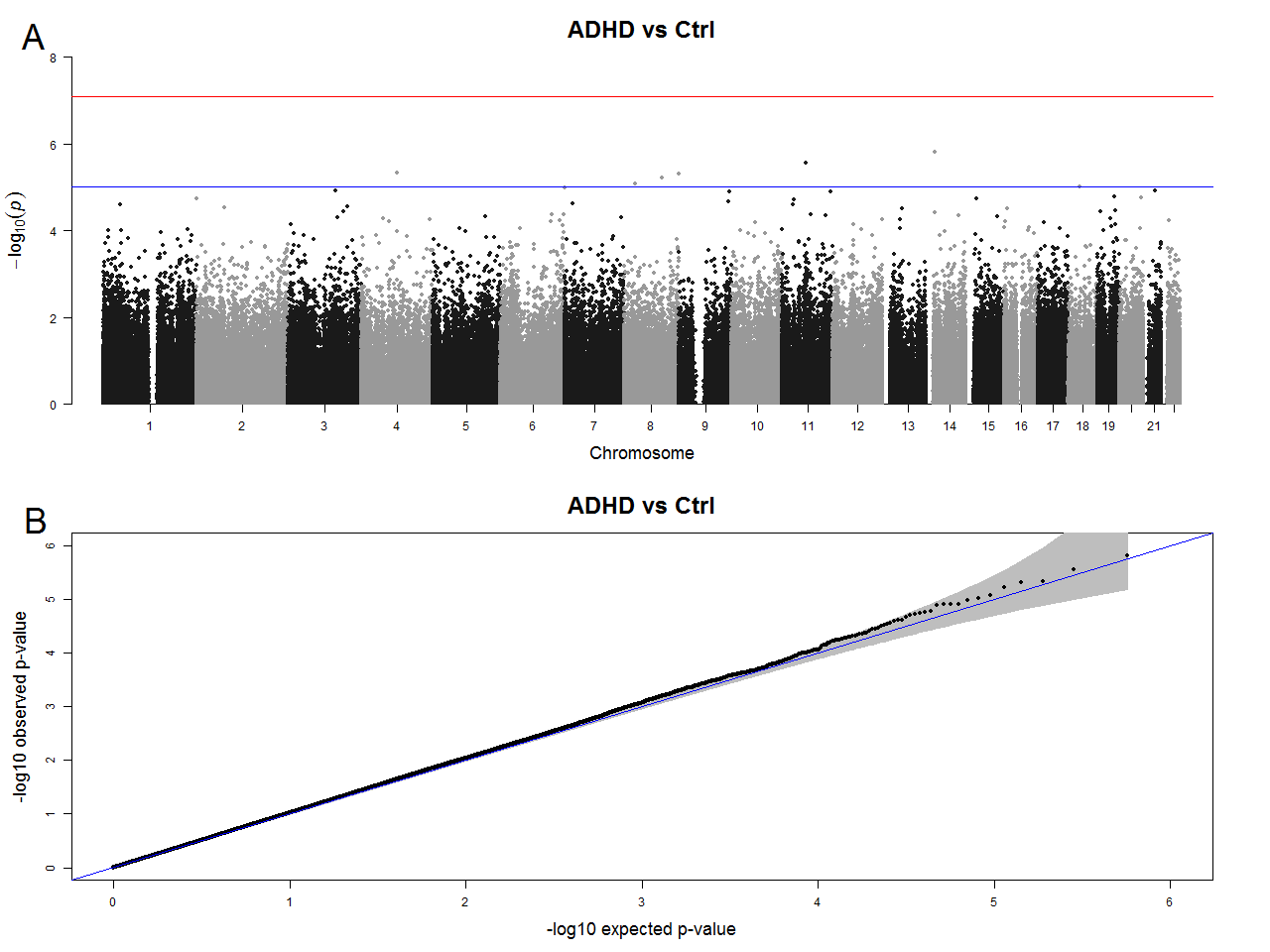


(A) A Manhattan plot for the EWAS of ADHD diagnosis. P-value thresholds indicated on the Manhattan plot are p=1e-5 (blue) and p=8.8e-8 (red). (B) A Q-Q plot for the EWAS of ADHD diagnosis.

Figure S6. Manhattan and Q-Q Plots for ADHD PRS


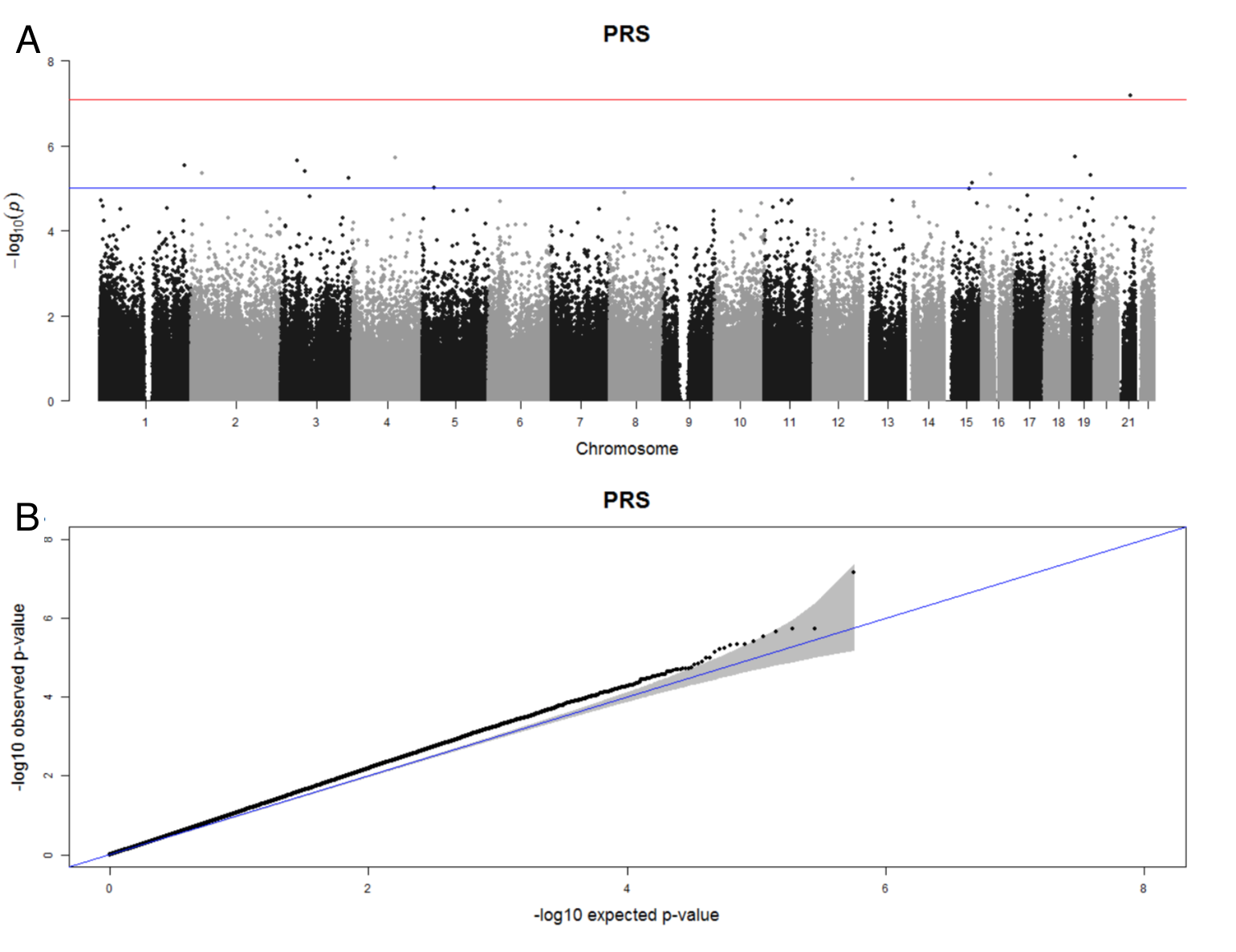


(A) A Manhattan plot for the EWAS of the ADHD PRS. P-value thresholds indicated on the Manhattan plot are p=1e-5 (blue) and p=8.8e-8 (red). (B) A Q-Q plot for the EWAS of the ADHD PRS.

Figure S7. Genomic locations and results of DMP and mQTL analyses.


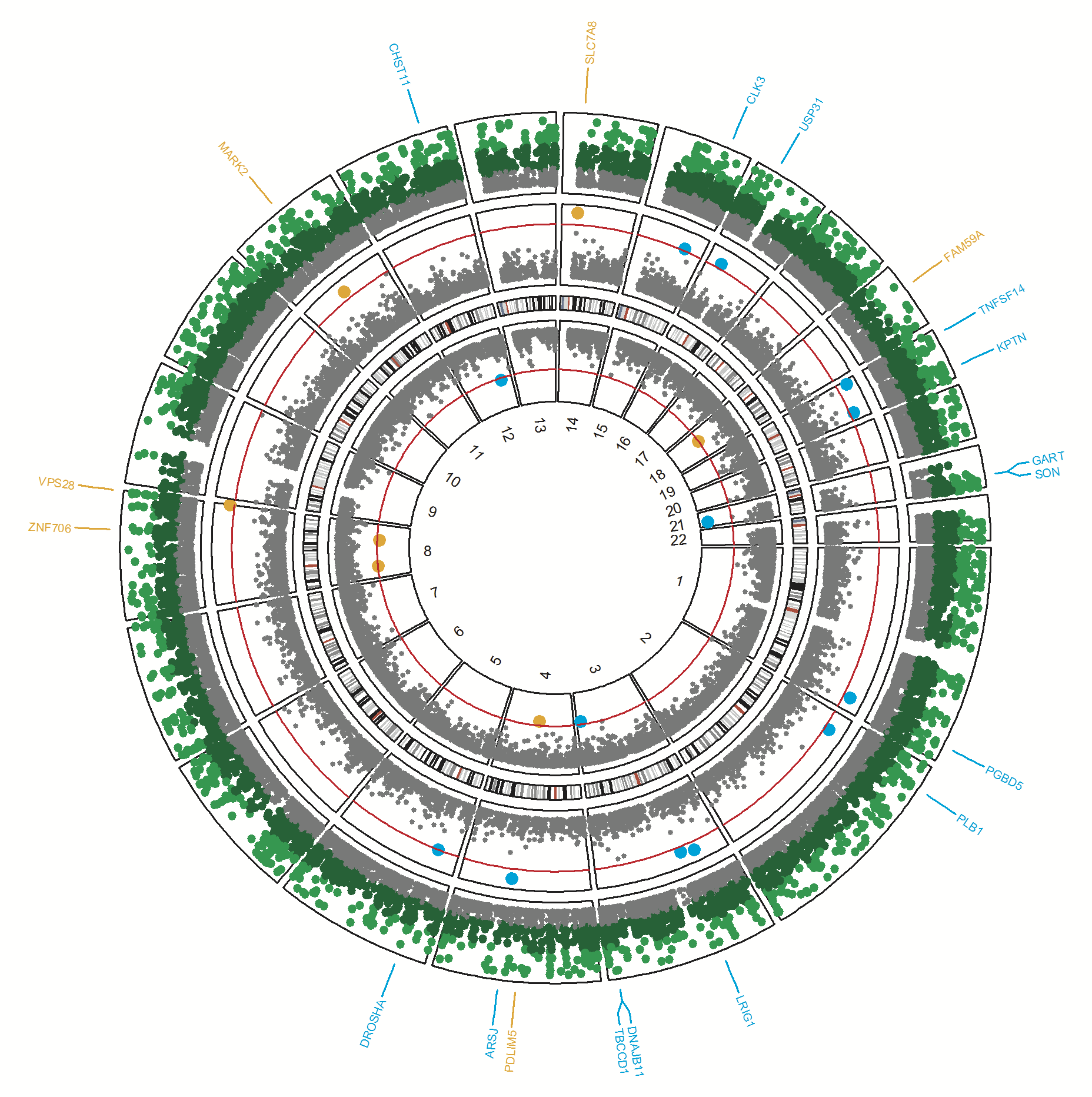


The outer track shows -log_10_ p-values of the most significant mQTL for each methylation probe, with the colors corresponding to the following p-value thresholds: light green < 1e-200 < dark green < 1e-100 < gray < 1e-25. The middle and inner tracks show the top DM probes (p<1e-5) in the ADHD vs. control (orange) and the ADHD PRS (blue) models, as well as probes with p-values > 1e-5 and < 0.05 in the ADHD vs. control model (gray). The middle track corresponds to probes positively correlated with ADHD status or the ADHD PRS. The inner track corresponds to probes negatively correlated with ADHD status or the ADHD PRS.

Figure S8. Differentially Methylated Region on Chromosome 6

For each sex, the average methylation value is shown for subjects with low ADHD PRS (scores in the 1^st^ quintile) vs. high ADHD PRS (scores in the 5^th^ quintile), at all 8 probes in the DMR on chromosome 6. Across the DMR, females with high PRS (green line) have significantly higher methylation values than females with low PRS (red line). The opposite relationship is true for males, although the effect size is much smaller.

Figure S9. Colocalization of mQTLs and ADHD-associated SNPs on Chromosome 12

GWAS (gray; top) and mQTL (red; center) results on 12q21. Methylation QTLs for which there is evidence of colocalization from the SMR method (and the probes involved) are highlighted at top.

Figure S10. Colocalization of mQTLs and ADHD-associated SNPs on Chromosome 15

GWAS (gray; top) and mQTL (red; center) results on 15q21. Methylation QTLs for which there is evidence of colocalization from the SMR method (and the probes involved) are highlighted at top.

Figure S11. Network of brain-expressed genes near ADHD PRS-associated DMPs.


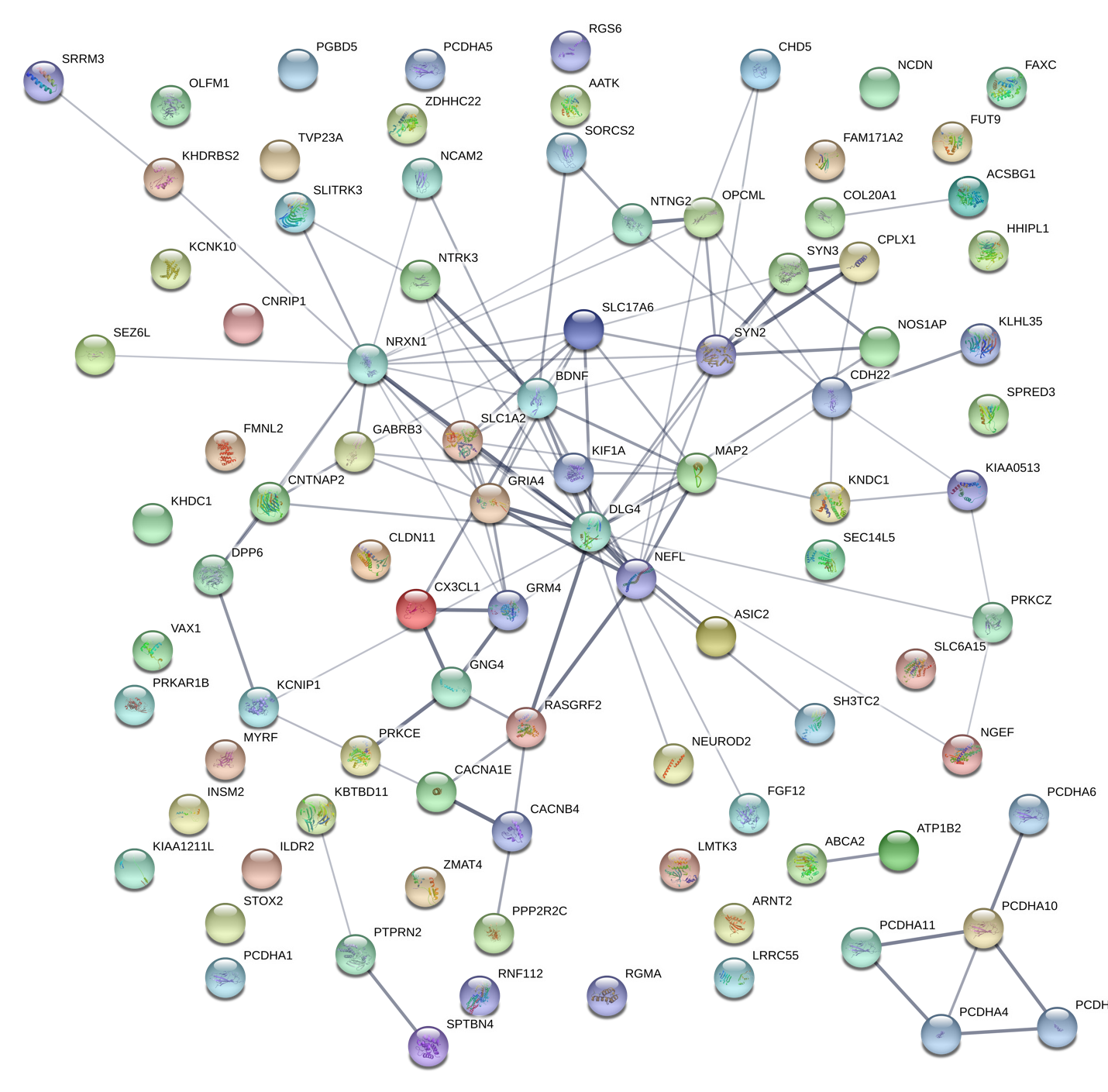


Protein-protein interactions among the 91 brain-expressed genes (defined by the Human Protein Atlas) that contain, or are near, ADHD PRS-associated DMPs (p ≤ 0.001). Protein interactions are all interactions with medium confidence (interaction score >= 0.4) from the STRING database (<https://string-db.org>); edge weight is proportional to interaction score.
